# The Chinese public’s awareness and attitudes toward genetically modified foods with different labeling

**DOI:** 10.1038/s41538-019-0049-5

**Published:** 2019-09-26

**Authors:** Yawei Zhao, Haiyan Deng, Changxin Yu, Ruifa Hu

**Affiliations:** School of Management and Economics, Beijing Institute of Technology, 5 South Zhongguancun Street, Beijing, 100081 China

**Keywords:** Science, technology and society, Economics

## Abstract

This paper analyzes the awareness and attitudes of the Chinese public toward genetically modified (GM) foods with different types of labeling and evaluates the impact of public confidence in the government management of GM food labeling has on their attitude. From 2015 to 2016, we conducted a series of surveys to collect data from 1730 respondents, which included consumers, farmers, media, and local agricultural officials in agricultural departments. The results show ~60% of the Chinese public do not know that they usually consume or purchase GM products or products containing GM ingredients. Nearly 80% of the Chinese public are accepting foods labeled as not containing GM ingredients, 57% are accepting foods without labeling, and ~40% are accepting GM-labeled foods. The respondents with a lack of confidence in the government are less likely to embrace GM foods. Those that are more aware of GM products are more likely to accept GM labeled foods. The group having the most positive attitude toward GM-labeled foods is the media, followed by agricultural officials, while the group having the most negative attitude toward GM labeled foods is farmers. Our findings provide an empirical basis to inform GM food labeling policy discussions and possible revisions, which may promote the development of GM foods in China.

## Introduction

Progress in the research, planting, and application of genetically modified organisms (GMOs) in China has been on-going for many years. China was one of the first countries to commercialize genetically modified (GM) crops and has made large investments in the research and development of GM technologies.^1,2^ The government has approved safety certificates for seven types of GM crops, though only insect-resistant cotton and disease-resistant papaya have achieved large-scale commercial production. In 2008, the State Council approved the establishment of major GM projects to support the research and development of agricultural GM technologies.^3^ A review of the scientific literature reveals Chinese researchers have cloned more than 100 important genes, obtained over 1000 patents, and have made numerous significant achievements in the research and development of GM technologies.^4^

In order to strengthen the management of GM food labeling and sales, while protecting consumers’ “right to know,” the Agricultural Genetically Modified Organisms Identification Management Measures were issued in 2002.^5^ According to this regulation, five types of GM crops within 17 categories of products must be mandatorily labeled. The five GM crops subject to labeling under this regulation include soybean, maize, cotton, rape, and tomato. Among the five types of crops that must be labeled, soybean includes soybean seeds, soybeans, soybean flour, soybean oil, and soybean meal; Corn includes corn seeds, corn, corn oil, and corn flour; Rape involves rapeseed for planting, rapeseeds, rapeseed oil, and rapeseed meal; Cotton includes cottonseed; Tomato includes tomato seed, fresh tomatoes, and tomato paste. Other products, not included in the regulation, may be voluntarily labeled or have no labeling requirement.^5^ For example, soybean oils produced and sold in China must indicate whether the raw soybeans include GM ingredients.^6^ Products such as GM papaya or products using imported GM sugar beet as raw material do not need to be labeled because these GM crop products are not included in the labeling regulation. GM tomato has not been approved for adoption in China, but the government had previously approved the commercial release of virus-resistant tomatoes.^7^ China has already approved GM cotton and GM papaya for commercial adoption, in addition to approving GM soybean, cotton, maize, rape, and sugar beet for import as raw materials for processing. So, tomato, maize, rape, and soybean are also subject to the 2002 labeling regulation.

Compared to other countries that commercialize GM products, e.g. Europe, the U.S. or Japan, the labeling system in China is relatively strict.^8^ There are three methods for identification of GM products in China, “Transgenic XX,” “Transgenic XX processed products,” and “the raw materials for processing of this product contain transposed XX, but the product does not contain GMOs”.^5^ When agricultural GMOs are difficult to identify by packaging or labeling, the following methods can be used to label the products: (a) marked on the product display; (b) marked on the price tag; (c) set up a signboard; (d) marked on the container; and (e) the sellers declaring that the products are GMOs in an appropriate manner. If imported agricultural GM products without packaging and labeling are difficult to mark with a signboard, their GM status should be indicated on the customs declaration.

The public’s attitude toward GM technologies and products, which is mediated by GM food labeling, may have a significant impact on government policy formulation and investment in new GM technologies. On the one hand, GM products have been labeled to satisfy consumers’ “right to know” and choose,^9^ potentially reducing asymmetric knowledge and understanding of GM technologies between producers and consumers,^10^ thereby increasing consumer acceptance. On the other hand, the intense debate on GM food labeling seems to have negatively influenced the public attitudes toward GM technologies and foods in China,^11^ which could further impact national decisions to develop and commercialize GM crops.^12,13^ From 2002 to 2012, the proportion of Chinese consumers who considered GM foods unsafe for consumption increased from 13 to 45%.^14^ And, despite the increase in consumption of GM soybean in China, Chinese consumers still lack knowledge and understanding of GM technologies,^15,16^ which further suggests that the current mandatory GM labeling policy in China might negatively influence the Chinese public’s attitude toward GM foods. Thus, research on the public attitudes toward GM foods with labeling will not only contribute to the refinement of labeling policies, but also help to elevate and improve public discussions concerning the sustainability of GM crops and safety of GM foods.

Related studies have shown that public trust in government management of GM labeling and safety assessments likely affects individuals’ attitudes toward GM labeled foods. For instance, Kikulwe et al.^9^ found that consumers who have more confidence in government institutions may have a 14% higher trust in GM food labeling. However, other studies conclude that individuals who believe the government implements a mandatory labeling policy for GM food are less likely to consume GM foods.^17^ Therefore, a mandatory GM food labeling policy implemented by the government may not generate trust from consumers and could result in a lack of willingness to purchase GM foods.^9^

Although there have been many studies on public attitudes toward GM foods in China, this study is the first to document public attitudes toward GM foods with different types of labeling and the determinants of these attitudes across four distinct public groups. Previous studies have focused on the general attitudes of specific groups toward GM technologies and the influence of GM food labels on consumer behavior. For example, Deng et al.^18^ proved that most Chinese agribusiness executives are worried about GM foods and oppose GM crop adoption. Huang et al.^11^ indicated that nearly 75% of scientists support the development of agricultural GM technologies, but only 29% of scientists are willing to buy GM soybean oil, which is similar to urban consumers (25%). Vecchione et al.^19^ found that GM food labeling would impact consumer buying decisions and Huffman et al.^20^ found that GM food labels reduced the price consumers were willing to pay for a product by ~14%. Meanwhile, new regulations on labels and safety tests are likely to lead to an increasing public awareness of the application of biotechnology in agricultural products.^21^

The objectives of this paper are to examine Chinese public awareness of GM food labeling, attitudes toward GM foods with different types of labeling and the impact of public confidence in the government’s management of GM food labeling on these attitudes. Our study’s population includes consumers, farmers, media, and local agricultural officials from county agricultural departments. The reasons we focus on these interest groups are as follows. First, consumers are direct buyers of GM foods, so their attitudes are important for both producers and policymakers. Second, given the large proportion of the agricultural population in China, farmers are not only the users of GM seed and producers of GM products, but also a major group of consumers of GM foods. Third, the media represents the tone or attitude on the coverage of GM foods, which may influence public opinion, so their attitudes toward GM food labeling might also have a significant impact on the final consumers’ attitudes. Finally, officials are the directors of agricultural department in the counties of different provinces. Their attitudes, to some extent, represent the government officials’ attitude toward GM food labeling. Therefore, it is necessary and important to explore these interest groups’ attitudes toward GM foods with different types of labeling.

## Results

### Awareness and attitudes toward GM food with different labeling in China

To capture public awareness, whether consumers know that the products they purchase or consume are GM products, and attitudes toward GM foods with different types of labeling, we choose foods with three types of labeling: (1) Foods without labeling, which does not indicate whether or not the food contains GM products, (2) Foods labeled as not containing GM ingredients, (3) Foods labeled as GM products, which includes GM edible oil, GM condiments and GM meat such as pork or chicken that has consumed GM feeds (since papaya has not been widely grown in China, we do not discuss it here). GM edible oil refers to edible oil made by GM soybeans or GM soybean ingredients while GM condiments mean products such as soy sauce or vinegar produced by GM soybeans or GM soybean ingredients. GM meat refers to livestock that have consumed GM feeds. We chose to examine these three product categories subject to GM food labeling because they exist or have been commonly found in the Chinese market. Edible oil and condiments produced by GM soybeans are the most commonly purchased and consumed GM products subject to GM food labeling. Pork and chicken are popular meat products in China and most of the compound feed used in Chinese livestock and poultry production contains GM ingredients.^22^ Although meat products that consumed GM feed do not need to be labeled based on the labeling regulation, China imports over 70 million tons of soybeans (over 90% are GMOs), and millions of tons of maize (sizable percentage of which are GMOs) in recent years.^23^ These soybeans and maize are mainly used for processing into animal feed and edible vegetable oil.^24^ Therefore, this study examines Chinese public awareness of these facts and attitudes toward GM edible oil, GM condiments, and meat products that have consumed GM feed, given their widespread consumption and presence in the marketplace.

The results in Table 1 show that the public has a low level of awareness of these three types of commonly consumed GM foods. In total, over 60% of respondents do not know that the foods that they usually buy or consume include GM ingredients, while <40% do know. Within the consumer interest group, 67% of people do not know that some livestock in China consumes GM feeds. Less than one third of consumers know condiments are made from GM soybeans. More consumers know soybean oil is made from GM soybeans. For farmers and the media, the percentage of those who know edible oil is made from GM ingredients is 52 and 54%, respectively. Surprisingly, a large proportion of agricultural officials do not know that meats and condiments are made from GM products, despite the fact that they have more access to agricultural knowledge and technology.

**Table 1:** Public’s awareness of GM foods (%)

	Total		Consumer		Farmer		Media		Agricultural Official	
	Know	Do not know	Know	Do not know	Know	Do not know	Know	Do not know	Know	Do not know
GM meat	33	67	33	67	43	57	41	59	25	75
GM oil	42	58	41	59	52	48	54	46	48	52
GM condiment	32	68	32	68	41	59	40	60	28	72
Average	36	64	35	65	45	55	45	55	34	66

Source: authors’ survey, 2015–2016

Notes: GM meat means livestock products such as pork and chicken fed by GM feeds; GM oil means edible oil produced by GM soybean; GM condiment are products such as soy sauce made by GM soybean

Public attitudes toward GM foods with different types of labeling are reported in Table 2. The overall results show that nearly 80% of the Chinese public accept foods that are labeled as containing no GM ingredients, 57% of the Chinese public accept foods with no labeling, and around 40% accept foods labeled as GM products. Of note, while 79% of consumers accept foods labeled as non-GM products, 57% accept foods without labeling, over 60% of consumers do not accept the three categories of GM labeled foods. However, the survey analysis did not reveal any differences in attitudes toward different types (meat, oil, and condiments) of labeled GM foods. These results are consistent with Amin et al.^25^ study which indicated that stakeholders’ attitudes do not vary among different types of GM products. But it does contradict with the conclusion of Burton et al.^26^ that UK consumers’ attitudes toward GM foods with modified plant genes and those with modified animal genes are significantly different. It should be noted that Burton’s study was comparing products from genetically modified animals, rather than animals that have consumed GM feed as was the case in this study.

**Table 2:** Public attitudes toward various GM food labels (%)

	Total		Consumer		Farmer		Media		Agricultural Official	
Attitudes	Accept	Oppose	Accept	Oppose	Accept	Oppose	Accept	Oppose	Accept	Oppose
GM foods with										
No labeling	57	43	57	43	33	67	74	26	52	48
Labeled non-GM	78	22	79	21	57	43	84	16	76	24
Labeled foods										
Labeled GM meat	38	62	35	65	19	81	76	24	56	44
Labeled GM oil	43	57	39	61	30	70	80	20	65	35
Labeled GM condiment	42	58	39	61	24	76	83	17	62	38
Average	41	59	38	62	24	76	80	20	61	39

Source: authors’ survey, 2015–2016

For farmers, 57% accept foods labeled as containing non-GM products, 33% accept foods without labeling and 24% accept GM-labeled foods. In contrast, most journalists hold positive attitudes toward GM products, with nearly 80% of the journalists accepting GM-labeled foods without preference toward how the products are labeled. We hypothesize that the mainstream media interest group surveyed had performed significant background research on GM technologies in order to ensure the objectivity and impartiality of their prior GMO reporting. Their foundational knowledge regarding GM technologies and products likely went beyond the basic bioscience knowledge assessed in this research survey and may explain the media’s more objective and positive opinion of GM technologies. This finding is consistent with our observation that fewer negative reports about GM technology by the Chinese mainstream media have occurred in recent years. The attitudes of government agricultural officials appear to be mostly positive as well, with acceptance rates of GM-labeled foods above 50%.

### Confidence in the government management of GM labeling

Another objective of this paper is to examine the Chinese public confidence in the government management of GM food labeling. The survey results in Table 3 show that over 50% of the Chinese public have confidence in the government management of GM food labeling. Particularly, 42% of consumers trust the government management of GM food labeling, while 51% do not and 7% have no knowledge on this matter. More than 50% of farmers are confident in government management, while 17% are not. Around one quarter of farmers have no knowledge on the subject. The media have the lowest level of confidence in the government management of GM food labeling. Specifically, only 34% have confidence in the government management of GM food labeling, 46% do not have confidence, and nearly 20% have no knowledge. Agricultural officials show the highest level of confidence in the government management of GM food labeling. Among them, 74% are confident, 11% are not, and the remaining 15% have no knowledge of this issue.

**Table 3:** Public confidence in the government management of GM food labeling (%)

	Consumers	Farmers	Media	Agricultural official
Confident	42	57	34	74
Unconfident	51	17	46	11
No knowledge	7	26	20	15

Source: authors’ survey, 2015–2016

### Determinants of attitudes toward GM food labeling

Estimates of the determinants of public attitudes toward GM food labeling are shown in Table 4. There are five dependent variables representing different labeling cases as well as a column indicating the trust in government management of GM food labeling. In the model of consumer confidence in government management of GM food labeling, the instrumental variable denoted by dummyiv, represents the public attitudes toward government management of the territorial dispute (see Methodology section 4 for more details). This has a significant effect on the confidence in the government management of GM food labeling. It should be noted that, in the bivariate probit (biprobit) model, the confidence-in- government variable equals 1 if individuals do not have confidence in the government, otherwise it equals 0. Therefore, the explanation of the coefficient of the confidence-in-government variable should be interpreted with care. If the coefficient is negative, it indicates that individuals who have no confidence in the government management of GM food labeling are less likely to accept GM foods. If the coefficient is positive, individuals who have confidence in government or do not have opinions on their confidence in the government are more likely to accept GM foods.

**Table 4:** Determinants of public attitude toward GM food labeling

Variables	Trust in government management	No label	No GM ingredients	GM meats	GM soybean oil	GM condiments
Trust in government	–	0.739 (0.525)	−0.766** (0.343)	−0.912* (0.499)	−0.817* (0.473)	−1.043** (0.452)
Awareness	–	−0.0289 (0.0569)	−0.103 (0.0652)	0.205*** (0.0674)	0.339*** (0.0652)	0.287*** (0.0701)
dummyiv	−0.321*** (0.0653)	–	–	–	–	–
Agricultural official	−1.371*** (0.157)	0.194 (0.308)	−0.556*** (0.186)	0.320 (0.303)	0.568** (0.284)	0.372 (0.295)
Farmer	−0.956*** (0.211)	−0.378 (0.316)	−0.834*** (0.192)	−0.837*** (0.244)	−0.518** (0.241)	−0.734*** (0.234)
Media	−0.235 (0.162)	0.516*** (0.167)	0.0666 (0.189)	1.043*** (0.218)	1.102*** (0.214)	1.171*** (0.245)
Knowledge	0.0450* (0.0234)	0.0367 (0.0238)	0.143*** (0.0259)	0.0145 (0.0236)	0.0170 (0.0235)	0.0131 (0.0233)
Female	0.0143 (0.0648)	−0.0204 (0.0621)	0.102 (0.0700)	−0.198*** (0.0677)	−0.187*** (0.0675)	−0.186*** (0.0677)
Age	0.00615** (0.00279)	−0.00124 (0.00292)	0.00521* (0.00316)	−0.0129*** (0.00364)	−0.0226*** (0.00377)	−0.0197*** (0.00406)
Bachelor below	−0.190* (0.0968)	0.275*** (0.0937)	0.210* (0.111)	0.0423 (0.102)	0.126 (0.104)	0.0410 (0.102)
Bachelor	0.0294 (0.0830)	0.0606 (0.0832)	0.136 (0.0882)	−0.0972 (0.0860)	−0.0216 (0.0857)	−0.0408 (0.0846)
Constant	−0.110 (0.141)	−0.339 (0.254)	0.521** (0.205)	0.544** (0.246)	0.799*** (0.230)	0.901*** (0.210)
Observations	1730	1730	1730	1730	1730	1730

Note: robust standard errors are between parentheses. ***p < 0.01, **p < 0.05, *p < 0.1

The results indicate that the confidence in the government management of GM food labeling has a significant impact on the dependent variables, except foods with no labeling. Those who do not trust the government management of GM food labeling are less likely to accept labeled GM products and foods labeled with containing no GM ingredients. This is probably because the people who are not confident in the government management of GM food labeling show less trust in the GM foods managed by the government. The lower levels of confidence in the government management of GM food labeling might also reflect public distrust of GMO safety assessment oversight by the government. This finding is consistent with evidence from previous research on consumer perceptions and trust in governance in China^14,27^ and other countries.^28,29^

The awareness of GM food labeling has a significant positive influence on consumers’ acceptance of GM labeled foods while having negative, but not significant, impact on foods without labeling and foods labeled as containing no GM ingredients. Results indicate that those who know that livestock is fed GM feeds or soybean oil/condiments are produced by GM soybeans are more likely to accept these GM products.

Moreover, the impact of consumers’ biotechnology knowledge about GM foods on the acceptance of GM labeled foods is not statistically significant, whereas the impact of consumer knowledge on the acceptance of foods labeled as GMO-free is significantly positive. The results are consistent with the results of Huang et al.^11^, which reveal that although agricultural scientists have more knowledge than consumers, the higher scientific knowledge does not result in a higher level of acceptance of GM foods. In addition, compared with male respondents, females are more opposed to GM labeled foods. The possible reason is that women, who usually buy foods in the market, are more concerned about food safety.^21^ Age has a significant negative impact on the acceptance of labeled GM foods while education level only has a significant positive effect on the acceptance of foods without labeling or foods with no GM ingredients. A possible explanation is that people who have a higher education may worry more about the perceived risks of GM foods.^27^

Public acceptance of foods with different types of labeling varies between interest groups examined in this survey. Compared with consumers, agricultural officials and the media have more positive attitudes toward GM labeled foods, while farmers have more negative attitudes. The relatively negative attitude of farmers may have several explanations. Farmers may believe that the mandatory GM labeling policy implemented by the government indicates that GM products are potentially risky, otherwise the government would not enforce such a strict labeling policy. Or, farmers lack of knowledge and awareness of GM technologies may make them more vulnerable to negative and false information about GM crops and products found on social media and the internet. These survey results are consistent with research on three African interest groups’ (farmers, consumers, and gatekeepers) attitudes toward GM foods, which also found that farmers have a lower awareness of GM technologies.^30^

## Discussion

This paper examines the determinants of Chinese public attitudes toward GM foods with different types of labeling among different interest groups and the effect of public confidence in the government management of GM food labeling on these attitudes. We conducted a series of surveys on four groups of respondents, namely consumers, farmers, media, and local government officials in agricultural departments. There are three different types of food labeling related to GM products, including foods with no labeling, foods labeled as GM products (meat labeled as having consumed GM feed, edible oil labeled as containing GM ingredients, and condiments labeled as containing GM soybean), and foods labeled as not having GM ingredients.

Overall, the Chinese public has a low level of awareness of common GM foods. Nearly 60% of respondents do not know that the foods that they usually buy or consume include GM ingredients. Most respondents prefer foods labeled as GMO-free and foods without labeling. Analysis of the survey data shows no significant difference in the attitudes toward labeled GM foods. The group holding the most positive attitudes toward GM foods is the media, followed by agricultural officials, while the group holding the most negative views toward GM foods are farmers.

Regression results show that those more familiar with GM products are more likely to accept labeled GM foods. The respondents lacking confidence in government oversight of GM labeling are less likely to accept GM products. Surprisingly, the effect of biotechnological knowledge on attitudes toward GM labeled food is not statistically significant, which is inconsistent with Cui’s study which concludes that consumers’ opinions of GM foods are related to their knowledge.^31^ One explanation for this result is that biotechnology knowledge may exert a smaller impact on public attitudes and behaviors than the misinformation disseminated by activists and other on social media in China.^32^ The public are often insensitive to scientific evidence but pay more attention to the risks, which could reduce the impact of knowledge. On the other hand, the questions measuring public biotechnology knowledge in our survey are more related to basic bioscience knowledge rather than GM technology, which might also influence the effect of knowledge on the attitudes.

Our research may be helpful in rethinking the implementation of mandatory labeling policy on approved GM products in China. It is widely accepted that mandatory food-labeling requirements are used to alleviate problems of asymmetric information and allow the consumers to have the “right to choose”.^33^ However, many studies suggest that the mandatory labeling policies do not provide consumers with valuable or useful information for decision making.^34,35^ Moreover, labeling requirement may increase the cost of products because of mandatory segregation systems along the food supply chain.^35^ In addition, a mandatory qualitative labeling policy requires effective and reliable detection techniques to detect traces of GM materials. So, policies may require huge efforts to validate tools for GMO analysis,^36^ which may further increase the cost of the products.

Therefore, the findings of this paper suggest that: (a) Regional agricultural officials need professional development or education on the science behind GM crops, such as safety studies, to bring them up to a level of competence on par with scientists in the bioscience field. Because the knowledge that they currently possess does not lead to purchasing behavior that differs from groups that likely know less about GM foods (consumers, farmers); (b) If the Chinese government intends to continue exploring GM technologies, widespread public education needs to occur to facilitate consumer acceptance and understanding; (c) The strict labeling regime may need some changes in order to increase acceptance of GM foods; and (d) Greater efforts should be made by the government to gain public trust.

Certainly, these survey results are not without limitations. Strong efforts were made to ensure collection of a representative survey sample, but results may only represent a portion of the general population due to sample size limitations. Therefore, if possible, we will increase the sample size of consumers in other provinces, farmers, media, and officials in further studies, which will support a more robust and persuasive analysis. In addition, the instrument variable included in the model may need to be refined for future work.

## Methods

### Model specification

Kikulwe et al.^9^ consider a conceptual model framework on consumers’ choices between foods labeled with GM information and those not labeled. Deng et al.^14^ provide a framework to test the impact of government trust on consumers’ attitudes toward GM foods. Following these models, we put forward a framework that models people’s attitudes toward GM foods with labeling. Its utility function is expressed as:

$${\mathrm{U}} = {\mathrm{U}}(L_{{\mathrm{NGM}}},L_{{\mathrm{GMP}}},L_{{\mathrm{GMS}}},L_{{\mathrm{GMV}}},L_{{\mathrm{GMN}}};C_{\mathrm{i}}),$$

where *L*_NGM_ represents foods labeled as non-GM foods, *L*_GMP_ meat such as pork and chicken fed GM feed and labeled as GM products, *L*_GMS_ soybean oil labeled as GM products, *L*_GMV_ vinegar or other condiments made from GM ingredients and labeled as GM products, *L*_GMN_ food labeled as not containing GM ingredients, and *C*_i_ the confidence in the government management of GM food labeling.

If *P*_NGM_, *P*_GMP_, *P*_GMS_, *P*_GMV_, and *P*_GMN_ stand for the prices of the respective foods above, *Y* is the budget of individuals, who maximize utility in the limit of this budget:

$$\begin{array}{l}{\mathrm{Max}}{\mathrm{U}} = {\mathrm{U}}(L_{{\mathrm{NGM}}},L_{{\mathrm{GMP}}},L_{{\mathrm{GMS}}},L_{{\mathrm{GMV}}},L_{{\mathrm{GMN}}};C_{\mathrm{i}}),\\ P_{{\mathrm{NGM}}}L_{{\mathrm{NGM}}} + P_{{\mathrm{GMP}}}L_{{\mathrm{GMP}}} + P_{{\mathrm{GMS}}}L_{{\mathrm{GMS}}} + P_{{\mathrm{GMV}}}L_{{\mathrm{GMV}}} + P_{{\mathrm{GMN}}}L_{{\mathrm{GMN}}} \le Y.\end{array}$$

To identify the determinants of Chinese public attitudes toward GM foods with different types of labeling, we propose an empirical model. Our focus is the determinants of public attitudes toward GM foods and the attitude differences among different groups. Based on previous studies and experience, we chose factors that can be identified and may have a significant impact on public attitudes. For instance, besides personal characteristics, we include public knowledge of biotechnology and awareness of GM foods. Moreover, we use four different groups of people for comparison. The general form of the model is formulated as:

$${\mathrm{Attitude}}_{\mathrm{i}} = f\left( {D_{\mathrm{i}},A_{\mathrm{i}},C_{\mathrm{i}}} \right) + \varepsilon _{\mathrm{i}}.$$

The dependent variable Attitude_*i*_ represents the attitudes toward GM foods with different types of labeling (acceptable or opposed). *D*_*i*_ represents personal characteristics, including age, gender (1 = female, 0 = male), education level, and bioscience knowledge. The education level is divided into three dummy variables: below bachelor, bachelor, or above bachelor. Knowledge is the number of correct answers for the five questions in the survey. *A*_i_ represents the public awareness of GM foods with labeling. *C*_i_ is the variable of confidence in the government management of GM food labeling. It may have endogenous issues because there might be unobserved variables in the attitude equation that not only influence public attitudes toward foods with different types of labeling, but are correlated with the variable public confidence in the government management of GM labeling. The bivariate probit model can apply to the model in which a dependent variable is a binary choice and an endogenous variable is also a binary choice. Given this, bivariate probit models are employed to estimate our models. The mixed-process regression model is as follows, and the estimated results are reported in Table 4.

$$\left\{ {\begin{array}{*{20}{c}} {{\mathrm{Attitude}}_{\mathrm{i}} = f_{\mathrm{i}}\left( {C_{\mathrm{i}},Group_{\mathrm{i}},D_{\mathrm{i}},A_{\mathrm{i}},\mu _{\mathrm{i}}} \right)} \\ {C_{\mathrm{i}} = f_{\mathrm{i}}\left( {{\mathrm{dummyiv}},{\mathrm{Group}}_{\mathrm{i}},D_{\mathrm{i}},A_{\mathrm{i}},\varepsilon _{\mathrm{i}}} \right)} \end{array}} \right.,$$

where dummyiv, the instrumental variable, represents the public attitude toward the government’s handling of the territorial dispute (Diaoyu islands) between China and Japan. This is a good instrumental variable because it theoretically reflects the public confidence in the government and is independent of agriculture, therefore not affecting public attitudes toward GM foods. Additionally, the regression results show that the instrumental variable is statistically significant.

### Data and descriptive statistics

To capture the different groups’ attitudes and awareness of GM foods with different types of labeling, we conducted a series of surveys during 2015–2016. All interviewers are graduate students on our team, who have received training on how to conduct research surveys.

The data on consumers were collected by face-to-face interviews during June–July 2015 in Beijing. Based on the different economic conditions and locations, we divided Beijing into three sections, that is, the inside ring, the middle ring and the outside ring, which represent the populations who live in the wealthier areas, the populations who live in relatively wealthy areas and those who live in relatively poor areas, respectively. Then we divided each section into four geographical directions (east, west, north, and south). Combining the rings and geographical directions, we obtained 12 sections of Beijing. In each section, we chose one big wet market and one big supermarket to ensure sample randomness. The reason that we choose consumers in the wet market and supermarket as representative consumers is that these people are usually the real decisionmakers. They purchase foods for their families, which likely includes GM foods. At each location, we randomly selected consumers who were either entering or leaving the market to respond to the questionnaire. A total of 1460 consumers were interviewed. For more details on consumers’ data, see Deng et al.^14^.

A series of surveys with farmers, who are presidents of agricultural cooperation communities (ACC), were conducted in 2016 coinciding with training of farming technology and practices in Beijing by the Chinese Ministry of Agriculture (MOA). The content of their training was cooperative management and policy. During all training rounds, we chose two groups of ACC farmers to complete the survey in order to capture their awareness and attitudes toward GM foods with different types of labeling. Fifty-four farmers were interviewed. Similarly, to understand government officials’ attitudes toward GM foods with labeling, surveys were conducted among directors of agricultural departments in the counties of different provinces in 2015, who visited Beijing for agricultural policy training. During the training, the government officials were organized by the MOA to help us complete the survey. As a result, a total of 146 agricultural officials from different counties in China were interviewed. In addition, from April to September 2015, we conducted a survey among journalists who had written reports about GM technologies and products in China. Of those surveyed, 70 journalists completed the questionnaire.

All respondents were asked to answer a series of questions relevant to GM food labeling. First, regarding the awareness of GM foods with different types of labeling (see the questionnaire in the appendix), the question “Do you know that, at present, most edible oils, condiments, and meat used in most restaurants are derived from GM plants or animals fed GM feeds?” (1=“know”; 2 = “do not know”) was asked. Second, a question about their attitude toward GM foods with labeling was included in the survey, “If, in accordance with the National Food Safety Law, restaurants have labeled their foods as containing GM ingredients, will you go to restaurants that use GM ingredients?” (1 = “yes”; 2 = “no”). To test the impact of government management confidence on Chinese public attitudes, we formulated the following question: “Currently, the National Food Safety Law requires that GM foods must be identified, do you think the government can do a good job in this area of regulation?” (1 = “yes”; 2 = “no”; 3 = “have no idea”). Additionally, respondents’ bioscience knowledge and their characteristics were also investigated.

The summary statistics of respondents’ characteristics are presented in Table 5. In total, 1730 respondents completed the survey, including 1460 consumers, 54 farmers, 70 journalists, and 146 agricultural officials. The response rate is around 75%, which means that 75% of the people who the graduate students tried to approach take the time to stop and answer questions. The average age of all respondents is 36. Most respondents are male (54%), but the male to female proportion is balanced. A total of 857 respondents hold a bachelor’s degree and 372 individuals hold a master’s degree or above, while 501 individuals only have a high school education or below. The highest education level in the farmer category is a bachelor’s degree, while medias’ lowest education level is a bachelor’s degree, which is reasonable because of the job’s nature and requirements. Furthermore, we also test Chinese public knowledge of GM technology with five common-sense questions about GMOs, mainly from Huang et al.^15^ and Huang et al.^11,15^ Respondents score one point for each correct answer, and zero otherwise. The individual total scores show that the media and agricultural officials have the highest average scores, while farmers score the lowest.

**Table 5:** Socio-demographic characteristics of respondents

	Total	Consumer	Farmer	Media	Agricultural official
Observations	1730	1460	54	70	146
Age	36	34	43	34	48
Gender					
Male	0.54	0.50	0.96	0.34	0.93
Female	0.46	0.50	0.04	0.66	0.07
Education (%)					
Below bachelor	29	31	63	0	10
Bachelor	50	48	37	37	80
Above bachelor	21	21	0	63	10
Knowledge	2.36	2.23	1.83	3.41	3.32

Source: authors’ survey, 2015–2016

Notes: the values for age and knowledge are averages. Knowledge is the average scores derived from five bioscience knowledge test questions

Statistical analysis: analysis of the survey results was finished by using the software program package – StataMP 14.

### Reporting summary

Further information on research design is available in the Nature Research Reporting Summary linked to this article.

## Supplementary information

Questionnaire

reporting summary

## Data Availability

A sample of the questionnaire, which has been translated into English, is available in supplementary information at npj: Science of Food’s website. The completed 1730 questionnaires and the resulting database for the statistical analyses are in Mandarin and are not publicly available, but can be made available from the corresponding author on reasonable request.

## Supplementary information

**Supplementary Information** accompanies the paper on the *npj Science of Food* website (10.1038/s41538-019-0049-5).
