# Supplementary material for: The Chinese public’s awareness and attitudes toward genetically modified foods with different labeling
Source: NPJ Sci Food. 2019 Sep 26;3:17. doi: 10.1038/s41538-019-0049-5 (PMC6763464; doi:10.1038/s41538-019-0049-5)
Supplement: Supplementary file 1 — Questionnaire [file 41538_2019_49_MOESM1_ESM.docx]

Public survey on genetically modified (GM) technology attitude and biotechnology policy

1. Currently, as the National Food Safety Law requires that all GM foods must be labeled, do you think the government can do a good job in this area of regulation?

1 = yes; 2 = no; 3 = have no idea

2. Do you know if, at present, edible oils, meat, and condiments used in most restaurants are derived from GM plants or animals fed GM feed?

| Genetically Modified Food | 1=know； 2=do not know |
| --- | --- |
| Soybean oil is made by GM soybean |  |
| Meat are from livestock fed by GM feeds |  |
| Condiments are made by GM soybean |  |

3. If, in accordance with the National Food Safety Law, the restaurants have labeled their foods as containing GM ingredients, will you go to the restaurants that use GM ingredients?

| Genetically Modified Food | 1=yes； 2=no |
| --- | --- |
| Food labeled as GM edible oil |  |
| Meat labeled as fed by GM feeds |  |
| Condiments labeled as containing GM soybean |  |
| No labeling |  |
| Labeled as not containing GM ingredients |  |

4. Please judge whether the following statement is correct? [ Fill in the corresponding space with “√” ]

| Statement | Right | Wrong | Have no idea |
| --- | --- | --- | --- |
| GM tomatoes contain genes, but ordinary tomatoes do not contain genes. |  |  |  |
| Genes change frequently during the life of humans and animals. |  |  |  |
| It is impossible to transfer animal genes into plants. |  |  |  |
| Hybrid rice is transgenic rice. |  |  |  |
| If the parents' blood type is A and B, their children's blood type may be O type. |  |  |  |

5. Do you think the way that the government handled with the Diaoyu Islands incident is appropriate?

1=too tough; 2= suitable; 3=too weak; 4=have no idea

6. Your personal basic information please

| Gender  0=male; 1=female | Age | Education level (The code is shown in the table below) |
| --- | --- | --- |
|  |  |  |
| **Instructions：**  Education level code：1=Doctor；2=Master；3=Bachelor；4=College degree；5= Secondary education；6= High school diploma；7= Junior high school education and below | | |

Investigator： Date of investigation：

Investigation site： Type of investigation：
